# Efficacy of Helicoverpa Armigera Nucleopolyhedrovirus on Soybean for Control of *Helicoverpa zea* (Boddie) (Lepidoptera: Noctuidae) in Arkansas Agriculture

**DOI:** 10.3390/insects13010091

**Published:** 2022-01-13

**Authors:** Joseph L. Black, Gus M. Lorenz, Aaron J. Cato, Nick R. Bateman, Nicholas J. Seiter

**Affiliations:** 1 Department of Entomology, Texas A & M University, College Station, TX 77843, USA; 2 Department of Entomology, The University of Arkansas, Lonoke Agricultural Center, Lonoke, AR 72086, USA; glorenz@uada.edu; 3 Department of Horticulture, The University of Arkansas, University of Arkansas Division of Agriculture, Little Rock, AR 72204, USA; acato@uada.edu; 4 Department of Entomology, The University of Arkansas, Rice Research and Extension Center, Stuttgart, AR 72160, USA; nbateman@uada.edu; 5 Department of Crop Sciences, University of Illinois, Urbana, IL 61801, USA; nseiter@illinois.edu

**Keywords:** nucleopolyhedrovirus, soybean, bio-pesticide, Heligen, *Helicoverpa zea*

## Abstract

**Simple Summary:**

*Helicoverpa zea* is a major pest of soybean, and has developed resistance to many insecticides. This has led to an exploration of alternative control tactics. One such tactic is Helicoverpa armigera nucleopolyhedrovirus (HearNPV). Although using a biological agent as a control tactic has limitations, the purpose of this study was to address the effectiveness of HearNPV in controlling each *H. zea* larval instar by simulating a field application on soybean and observing mortality. Furthermore, this study addresses the potential for infection in one generation to lead to infection in a sequential generation. Our results indicate that HearNPV is effective at controlling 1st–3rd instar larvae, but does not effectively control 4th or 5th instar larvae. Also, a sequential generation of 2nd instar larvae succumbed to viral infection faster than the previous infestation. Therefore, HearNPV is capable of cross-generational infection, and could be a useful tool in producer’s management tactics for *H. zea*.

**Abstract:**

Helicoverpa armigera nucleopolyhedrovirus (HearNPV) is a naturally occurring virus commercially produced for control of Heliothines, including *Helicoverpa zea*. One drawback with using this virus for control has been the slower time to mortality compared with synthetic insecticides. However, a new formulation (Heligen^®^) has anecdotally been thought to result in quicker mortality than previously observed. The objective of this study was to evaluate percent defoliation, the efficacy of HearNPV on mortality for each *H. zea* larval instar, and the potential for control of a second infestation. Fourteen days after the first infestation, all plants were re-infested with a second instar larva to simulate a second infestation. Helicoverpa armigera nucleopolyhedrovirus was effective at killing 1st–3rd instars, resulting in 99% mortality over 4–6 days. However, 4th and 5th instar mortality only reached 35%. Second infestation larvae died between 3.4 and 3.8 days, significantly faster than the 1st infestation of 2nd instars, which had a mean time to mortality of 4.9 days. An increase in mortality rate is probably due to increasing viral concentrations after viral replication within the first hosts. Final defoliation percentages were significantly smaller in the treated plants versus the untreated plants. Only 3rd and 4th instar larvae caused percent defoliation to exceed the current Arkansas action threshold of 40%. Helicoverpa armigera nucleopolyhedrovirus in the Heligen formulation can control 1st–3rd instars within 4–6 days, while keeping defoliation below the action threshold of 40%.

## 1. Introduction

Helicoverpa armigera nucleopolyhedrovirus (HearNPV) is a viral biopesticide specific to Heliothines, such as *Helicoverpa zea* (Boddie) (Lepidoptera: Noctuidae), the most damaging pest of soybean (*Glycine max* (L.) Merrill) in the mid–south United States [1,2]. HearNPV can be applied using conventional pesticide application equipment and is commercially available as Heligen^®^ (Agbitech LLC., Ft. Worth, TX, USA), Gemstar^®^ (Certis USA LLC., Columbia, MD, USA), or Helicovex^®^ (Corteva Agriscience, Wilmington, DE, USA). Nucleopolyhedrovirus formulations are relatively inexpensive compared with many of the synthetic insecticides, have no known off-target effects, and have the potential to induce epizootic events through horizontal transmission [3,4,5,6]. However, NPVs take a longer time to induce mortality, relative to conventional insecticides, and tend to suppress, not eradicate, the local pest population. HearNPV was first marketed as Elcar^®^ and multiple studies were conducted to evaluate its suitability in commercial agriculture, but ultimately, due to the marketed release of the Pyrethroid insecticide class in 1977, Elcar^®^ was not successfully implemented [7]. Earlier studies investigating Elcar^®^ found time to mortality ranged from 3 to 8 days [8,9,10,11]. This delay from application to mortality could discourage growers from implementing HearNPV into their spray regimes. Growers and consultants familiar with synthetic insecticides would typically expect eradication of the pest to occur within 3 days and suspect a failed application if live insects are observed beyond 5 days. The observed virulence of Heligen and other new formulations of HearNPV, coupled with developing resistance to insecticide classes in *H. zea* [12,13,14], suggest that HearNPV could be commercially viable, as it appears to prevent feeding and causes high levels of mortality [11,15].

Horizontal transmission is an important factor in the efficacy of HearNPV as an insecticide, as it is a critical part of inducing epizootic events [3,4]. Along with increasing viral infections in a localized area, horizontal transmission of HearNPV may lead to viral infection of subsequent infestations [16,17,18,19]. Although previous studies found the potential for Elcar^®^ to be transmitted from one infestation to the next at around 11% [20], there is a need to study the current commercially available products (Heligen^®^) and their potential to infect subsequent infestations.

The first objective of this study was to determine the ability of HearNPV, applied at the commercially recommended rate of 8.76 × 10^11^ OB/ha (116.8 mL Heligen^®^/ha), to induce mortality in the different instars of *H. zea*. The second objective of this study was to determine whether significant survival differences or delays in pupation were observed for each larval instar of *H. zea* when reared on soybean plants treated with HearNPV. The third objective of this study was to determine if an application of HearNPV would lead to a reduction in soybean defoliation by the infected larvae. The fourth objective of this study was to understand the cross-generational infectivity of HearNPV by simulating a second infestation following termination of the first infestation. We hypothesized that later instars would exhibit decreased mortality, with earlier instars dying faster than later instars. This would result in less defoliation in the earlier instars when compared with the untreated check, but not the later instars. In addition, we expected that the Heligen^®^ strain of HearNPV would be capable of infecting a simulated second infestation, regardless of the initial larval instar.

## 2. Materials and Methods

Soybeans were planted 1 May, 13 June, and 14 July, 2017, in 10.2 cm × 10.2 cm × 8.9 cm pots (Greenhouse Megastore, Danville, IL, USA) using potting soil (Scotts Miracle-Gro Company—Landscaping, Marysville, OH, USA). The soybean cultivar used was Pioneer 47T36 (DuPont Pioneer, Johnston, IA, USA) with no seed treatment. Pioneer 47T36 is glyphosate tolerant, which does not contain *Bt* technology. Soybeans were kept in a greenhouse at the Lonoke County Research and Extension Center in Lonoke, AR, where the temperature was maintained between 22.5° and 33.3 °C and a natural light photoperiod was utilized. Soybeans were watered twice daily until they reached the V3 growth stage [21], at which time the initial HearNPV efficacy experiment was initiated. *Helicoverpa zea* larvae utilized in this experiment were purchased from Benzon Research Inc. (Carlisle, PA, USA), and were physiologically staged to instar based on descriptions reported in Neunzig [22,23]. Plants randomly assigned to be untreated were caged and infested with a single larva before the treated plants were sprayed. Plants randomly assigned to be treated with HearNPV were removed for a short period of time to receive an application of Heligen at a rate equivalent to the recommended commercial rate of 8.76 × 10^11^ OB/ha (116.8 mL/ha) immediately before infestation (AgBiTech Corporation, Queensland, Australia). Heligen was applied with a CO_2_ backpack sprayer at 93.6 L/ha, using a ground speed of 4.8 km/hr. Treated plants were returned to the greenhouse after the application had dried. A single larva was placed on each soybean plant and then caged with a white insect rearing cage, 20 × 40 cm (BioQuip Products, Rancho Dominques, CA, USA). Cages were utilized to restrict the larvae from moving to other plants, localize defoliation, and restrict viral movement, as well as to eliminate potential cannibalism.

A randomized complete block design with two blocking factors was utilized to determine the ability of HearNPV to induce mortality in *H. zea* larvae feeding on soybean plants. The treatment arrangement was a 5 × 2 full factorial, with two factors: larval instar (1st−5th) and application of HearNPV (sprayed or unsprayed). The 2 blocking factors were location within the greenhouse (18 locations) and the run number (3 runs). Each run contained all combinations of both factors and was completed in 18 locations within the greenhouse. Each area consisted of 5 samples, for a total of 90 samples per run, with 60 of these samples being treated and 30 untreated samples. There were 12 replications for each treated larval instar and 6 replications for each untreated larval instar during each run, which resulted in 36 replications of each HearNPV-exposed larval instar and 18 replications of each unexposed larval instar. This design was utilized because the untreated plants were to verify no viral movement and to have a baseline of percent defoliation. In the first run, HearNPV spread to several untreated larvae, which were removed from analysis; therefore, wooden barriers 0.9 m × 1.5 m were used to separate the untreated from the treated plants for the two remaining runs. Time to pupation or mortality and percent defoliation were recorded twice daily from the initiation of each experimental run until termination. Percent defoliation was estimated using a pictorial sheet representing a soybean leaf at different defoliation percentages, and cumulative maximum percent defoliation was reported.

The cross-generational infectivity of HearNPV experiment was initiated using the same soybean plants from the previously described experiment. A duration of 14 days after the initiation of the initial HearNPV efficacy experiment, once all larvae from the experiment either died or pupated, each sleeve cage was re-infested with an uninfected 2nd instar larva to determine the cross-generational infectivity of HearNPV. For the purpose of this study, cross-generational infectivity refers to the ability of HearNPV to move across time from one larval population to the next and induce mortality, and treatments are distinguished based on the initial infestation larval instar. The same monitoring protocol was used as that in the first experiment.

Data concerning the final fate of each larva for all experiments—pupation or mortality—were subject to an ANOVA (*α* = 0.05), and a Fisher’s exact test, as the response variable was considered categorical. Fixed effects consisted of larval instar, viral application, and interaction effects between larval instar and viral application. Block number and run number were analyzed as random effects. To determine differences in the time to mortality, Kaplan–Meier survival curves were established followed by the log-rank test. Cumulative maximum defoliation percentages were subjected to an ANOVA (*α* = 0.05), followed by Tukey’s post hoc analysis. Mortality of the second infestation was used to develop Kaplan–Meier survival curves, where differences were tested by the log-rank test. Differences in survival time between the initial and second infestation were determined using an ANOVA (*α* = 0.05), followed by Tukey’s post hoc analysis. All data subject to ANOVAs were analyzed using R Studio (R Core Team 2020), and all nonparametric survival analysis occurred in JMP 15.

## 3. Results

**Figure 1:**
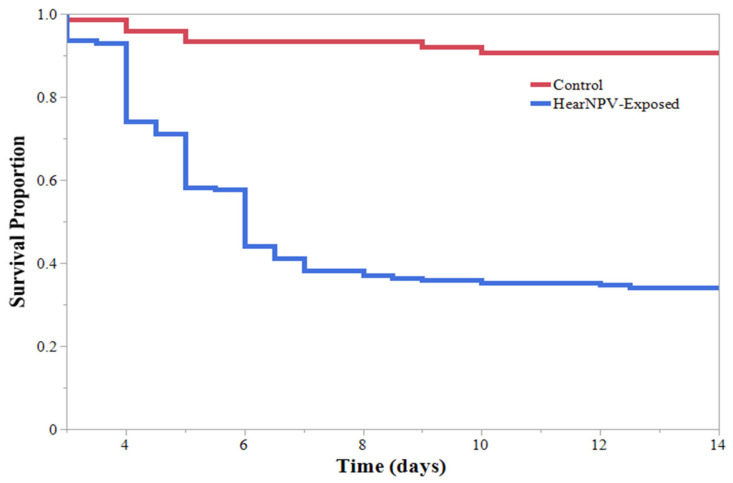
*Helicoverpa zea* (Lepidoptera: Noctuidae) Kaplan–Meier survival curves for all larvae of the initial infestation feeding on sprayed and unsprayed V3 soybeans, not accounting for differences in larval instars. Log-rank test: χ^2^ = 59.032, *p* < 0.0001.

**Figure 2:**
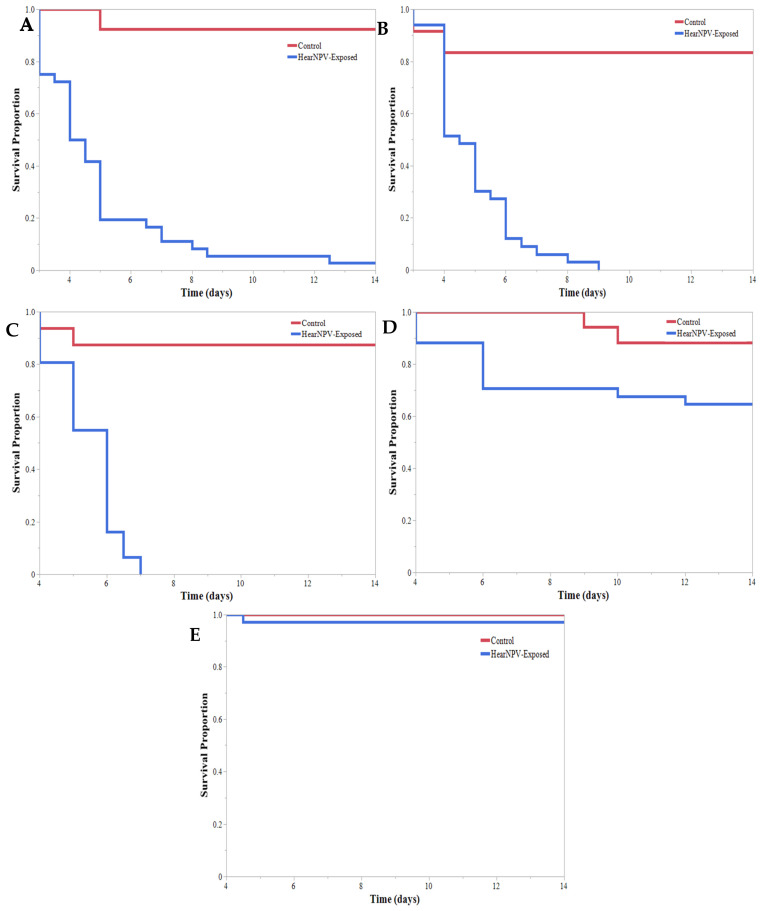
*Helicoverpa zea* (Lepidoptera: Noctuidae) Kaplan–Meier survival curves for each larval instar reared on Helicoverpa armigera nucleopolyhedrovirus-sprayed and unsprayed V3 soybeans. (**A**) Significantly different survival curves for 1st instar larvae reared on unsprayed and virus-sprayed soybeans. Log-rank test: χ^2^ = 30.924, *p* < 0.0001. (**B**) Significantly different survival curves for 2nd instar larvae reared on unsprayed and virus-sprayed soybeans. Log-rank test: χ^2^ = 24.445, *p* < 0.0001. (**C**) Significantly different survival curves for 3rd instar larvae reared on unsprayed and virus-sprayed soybeans. Log-rank test: χ^2^ = 30.566, *p* < 0.0001. (**D**) Survival curves for 4th instar larvae reared on unsprayed and virus-sprayed soybeans were not significantly different. Log-rank test: χ^2^ = 7.249, *p* = 0.0071. (**E**) Survival curves for 5th instar larvae reared on unsprayed and virus-sprayed soybeans. Log-rank test: χ^2^ = 0.4722, *p* = 0.492.

**Figure 3:**
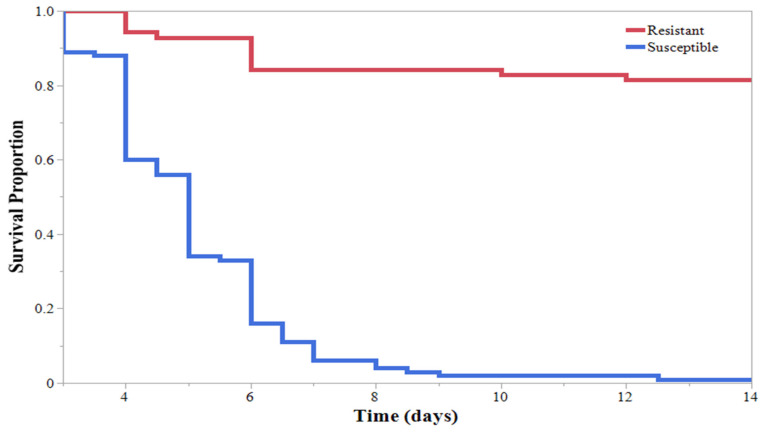
Kaplan–Meier survival curves for *Helicoverpa zea* (Lepidoptera: Noctuidae) larvae grouped into “Resistant” (4th and 5th instars) and “Susceptible” (1st, 2nd, and 3rd instars), reared on V3 soybeans sprayed with Helicoverpa armigera nucleopolyhedrovirus. Significant differences were observed between survival curves. Log-rank test: χ^2^ = 123.89, *p* < 0.0001.

During the initial HearNPV efficacy experiment, larvae feeding on virus-treated soybeans had a significantly different Kaplan–Meier survival curve than larvae feeding on untreated soybeans (Figure 1). Within the HearNPV-sprayed treatments, survival curves were significantly different based on larval instar (Figure 2). The 1st, 2nd, and 3rd larval instars had the highest percentage of mortality (Table 1); only 1 of the 1st instar larva survived to pupation (Figure 2). All larval instars exhibited a significant decrease in survival when reared on virus-treated soybeans compared with their conspecifics in the untreated group, except the 5th instars, where no differences were observed (Figure 2). Time to mortality ranged from 4.5 to 6 days across all instars; however, only 1 of the 5th instars and 35% of 4th instars succumbed to a viral infection, compared with almost 100% of the 1st, 2nd, and 3rd instar larvae (Table 1). Therefore, instars were grouped into “Resistant” (4th and 5th instars) and “Susceptible” (1st, 2nd, and 3rd instars), and Kaplan–Meier survival curves were established showing a significant decrease in survival for the “Susceptible” group compared with the “Resistant” group (Figure 3). This indicates that an application of HearNPV should target the “Susceptible” group rather than the “Resistant” group.

**Figure 4:**
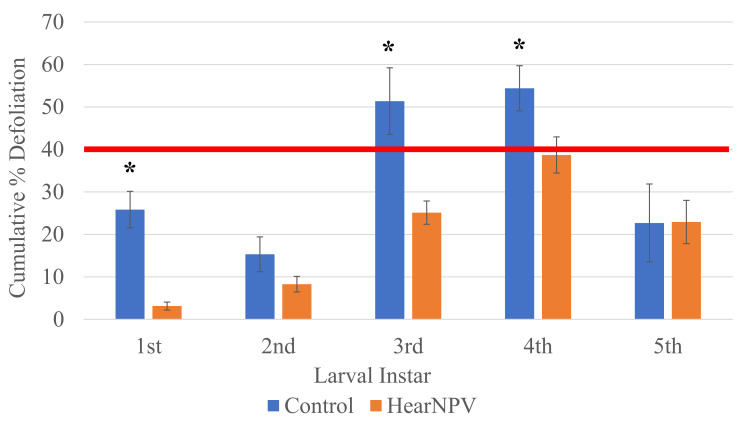
Determining the efficacy of Helicoverpa armigera nucleopolyhedrovirus versus the untreated through percent defoliation across *Helicoverpa zea* (Lepidoptera: Noctuidae) larval instar when applied to V3 soybeans compared with the Arkansas economic threshold of 40%. * Denotes significant differences for that instar across treatments using ANOVA (α = 0.05) and Tukey’s post hoc analysis (*p* = 0.05). The red line denotes the Arkansas action threshold of 40% defoliation.

Cumulative maximum defoliation percentages were significantly smaller in the virus-treated soybeans infested with 1st, 3rd, or 4th instar larvae, compared with the untreated conspecifics. There was no significant difference between sprayed and unsprayed plants in cumulative maximum defoliation for 2nd instar or 5th instar larvae. Unsprayed plant average defoliation ranged from 15% to 54%, with an average final defoliation of 35.4% across all instars (Figure 4), while sprayed plant average defoliation ranged from 3% to 39%, with an average final defoliation of 19.5%. Plants sprayed with HearNPV had significantly less (*p* ≤ 0.001) defoliation than unsprayed plants (Figure 4). In both sprayed and unsprayed treatments, the 4th instar larvae caused significantly more defoliation than all other instars except the 3rd instar larvae (Figure 4). The 1st, 2nd and 5th instar larvae on unsprayed plants exhibited defoliation percentages of 26%, 15%, and 23%, respectively, with all but the 1st instars being significantly lower than 3rd instar larvae at 51%. Of the larvae on virus-treated plants, the plants infested with 3rd and 4th instar larvae had significantly more defoliation than all other larval instars except 5th instar larvae, but exhibited significantly less defoliation than was seen in conspecifics on unsprayed plants (Figure 4). Sprayed plants infested with 1st and 2nd instars had significantly less defoliation than all other treatments, but did not differ between each other. On unsprayed treatments, plants infested with 3rd and 4th instar larvae had defoliation percentages above the action threshold, while only virus-treated plants infested with 4th instar larvae had defoliation percentages close to the threshold (Figure 4).

**Figure 5:**
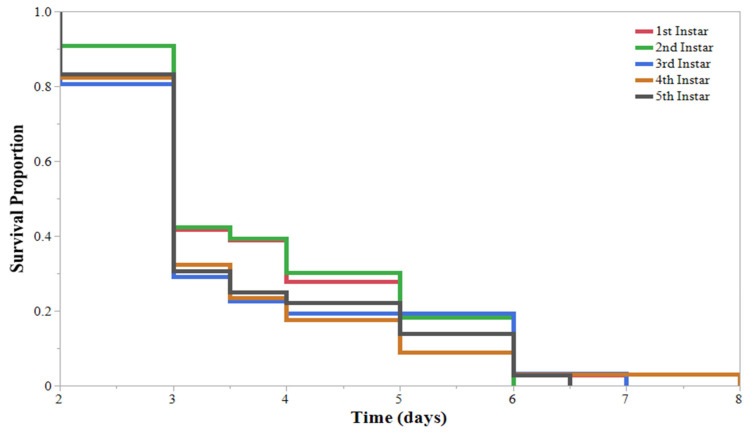
Kaplan–Meier survival curves for the 2nd infestation consisting of only 2nd instar larvae. Treatments are distinguished by the initial larval instar of the 1st infestation. There were no significant differences observed between survival curves. Log-rank test: χ^2^ = 1.192, *p* = 0.879.

**Figure 6:**
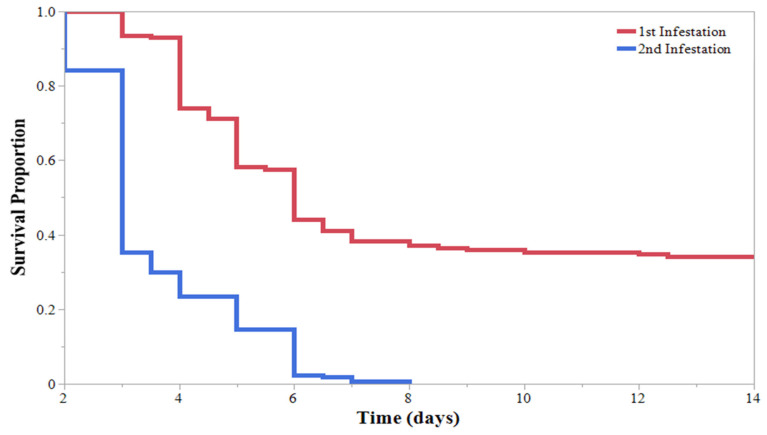
Kaplan–Meier survival curves for *Helicoverpa zea* (Lepidoptera: Noctuidae) larvae comparing survival times of the 1st infestation and the 2nd infestation regardless of initial larval instar. The larvae from the 2nd infestation died significantly faster than larvae from the 1st infestation. Log-rank test: χ^2^ = 151.375, *p* < 0.0001.

**Figure 7:**
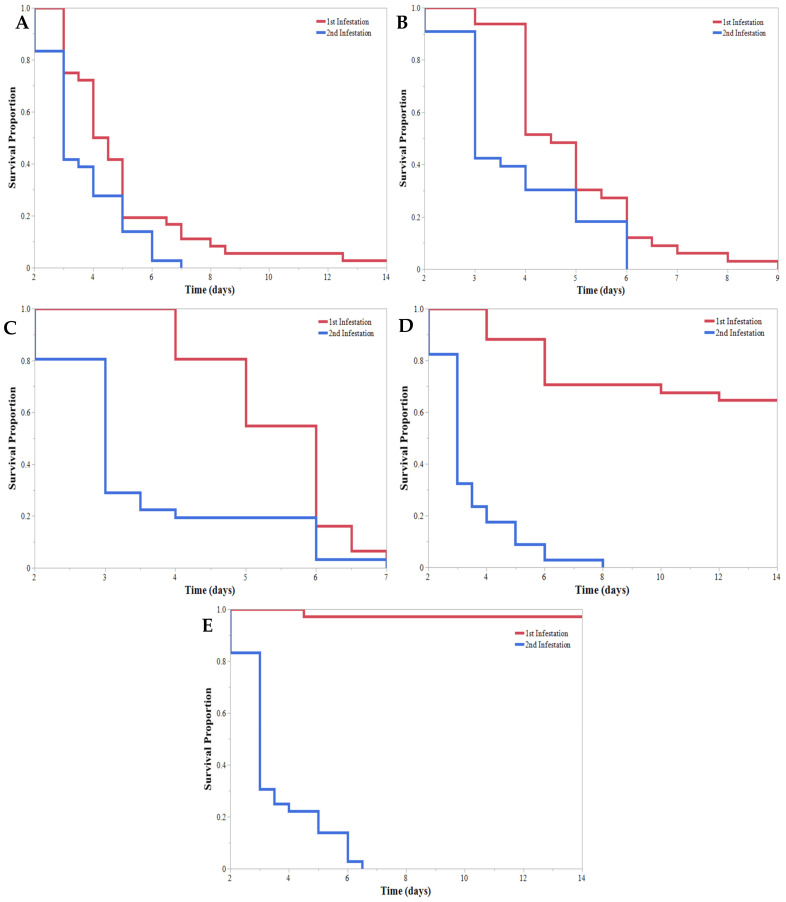
Kaplan–Meier survival curves comparing the different times to mortality for the 1st infestation and the 2nd infestation of *Helicoverpa zea* (Lepidoptera: Noctuidae) on V3 soybeans sprayed with Helicoverpa armigera nucleopolyhedrovirus. (**A**) The 1st instar larvae from the 1st infestation survived significantly longer than 2nd instar larvae from the 2nd infestation. Log-rank test: χ^2^ = 6.916, *p* = 0.0085. (**B**) The 2nd instar larvae from the 1st infestation survived significantly longer than 2nd instar larvae from the 2nd infestation. Log-rank test: χ^2^ = 8.081, *p* = 0.0045. (**C**) The 3rd instar larvae from the 1st infestation survived significantly longer than 2nd instar larvae from the 2nd infestation. Log-rank test: χ^2^ = 17.869, *p* < 0.0001. (**D**) The 4th instar larvae from the 1st infestation survived significantly longer than 2nd instar larvae from the 2nd infestation. Log-rank test: χ^2^ = 60.032, *p* < 0.0001. (**E**) The 5th instar larvae from the 1st infestation survived significantly longer than 2nd instar larvae from the 2nd infestation. Log-rank test: χ^2^ = 79.544, *p* < 0.0001.

A duration of 14 days after the initial application of HearNPV and infestation of *H. zea*, all samples were re-infested with a simulated 2nd infestation of 2nd instar *H. zea* larvae, initiating the cross-generational infectivity experiment. Treatments in this experiment were distinguished based on larval instar of the previous infestation (1st–5th). When added to previously sprayed plants, the reinfestation resulted in 100% mortality (Figure 5). The survival curves for the reinfestation did not significantly differ based on the initial larval instar of the first infestation (Figure 5). Larvae of the second infestation succumbed to HearNPV infection significantly faster than larvae of the first infestation (Figure 6). When comparing survival curves within a treatment but across simulated infestations, all survival curves of the reinfestation resulted in significantly faster times to mortality compared with the first infestation (Figure 7); therefore, the second infestation succumbed to HearNPV infection significantly faster than the first infestation.

## 4. Discussion

The HearNPV application caused mortality to occur within 4.5 and 5.5 days for the 1st–3rd instar *H. zea* larvae. The mean mortality time was 5 days. In the 2nd infestation, the average time to mortality was 3.5 days across the sprayed plants, regardless of previous larval instar. This ability to infect multiple infestations and continue to replicate in hosts could be of high value to growers and producers that typically rely on a “residual time”. There was a significant decrease in the mortality time in the second infestation compared with the first infestation. This could indicate an increase in virulence between infestations, perhaps due to the highly concentrated release of viral occlusion bodies in the rupturing of the infected larva’s cuticle [24]. Furthermore, as seen in Figure 7, HearNPV survived on plants containing a “resistant” host (4th and 5th instar larvae) and viral particles were still able to infect “susceptible” 2nd instar larvae during the second infestation, inducing mortality at a faster rate than the initial application. This suggests some viral replication occurred even in the “resistant” population.

An application of HearNPV reduced the amount of defoliation caused by all instars. This is likely, since the HearNPV killed the larvae before they are able to finish their larval life, rather than appetite suppression, since most mortality occurred in earlier instars, before later instars, where the majority of consumption occurs, were reached [25,26]. Across treatments there was a trend for the later instars to cause increased defoliation; however, 5th instar larvae were observed to feed significantly less than the 4th instars. It is possible that 5th instar larvae consumed the majority of their needed diet for pupation prior to the initiation of the study, which is supported by the rapid onset of pupation in 5th instar larvae (Table 1). When comparing defoliation percentages by instar across treatments, there was a significant reduction in defoliation for all instars except the 5th instar, when an application of HearNPV was made. Unsprayed plants that were infested were expected to have defoliation percentages above 40%; however, this did not occur. The 3rd and 4th instar larvae exhibited little mortality and caused over 50% defoliation. When contrasted to the sprayed treatment, only 4th instar larvae were close to 40% defoliation. Taken together, the results suggest that the target population for control in a soybean field should be mainly 1st−3rd instar larvae, as 4th instar larvae could still cause significant defoliation before death.

An application of HearNPV provided control for 1st, 2nd, and 3rd instar larvae, resulting in a mean mortality time of 5 days after application. Based on this mortality time and the decrease in damage, we speculate that applying HearNPV in a field on a population of 1st, 2nd, and 3rd instar *H. zea* larvae will result in control consistent with synthetic insecticides within a 6-day span. Therefore, this biopesticides might not be readily implemented into spray regimes but, with further education surrounding other limitations and benefits [27,28,29,30,31], it could be a useful tool for soybean growers. However, HearNPV in the form of Heligen does not appear to provide adequate control of 4th or 5th instars before high levels of damage occurs. HearNPV can provide control of sequential infestations when adequate amounts of virus remain; however, it is likely that many environmental factors leading to degradation of HearNPV were not captured by this study. It was also found that the second infestation died faster than the first, probably due to a concentrated release of viral occlusion bodies following the mortality of the first infestation or by viral replication and shedding in the more “resistant” population. Future studies should explore cross-generational interactions and look to include other environmental factors not considered in this study.

## Figures and Tables

**Table 1:** Determining the efficacy of Helicoverpa armigera nucleopolyhedrovirus (HearNPV) by investigating mortality, pupation, and time to mortality/pupation in *Helicoverpa zea* (Boddie) (Lepidoptera: Noctuidae) larval instars when applied to V3 soybeans previously treated with HearNPV, and the time to mortality of a simulated 2nd infestation 14 days after the initial application (DAA).

Initial Instar	% Mortality *	% Pupated *	Mortality (DAA) **	Pupation (DAA) **	2nd Infestation Mortality (DAA) **
1	97 a	3 c	4.7 b	14 b	3.7 a
2	100 a	0 c	4.9 ab	-	3.8 a
3	100 a	0 c	5.5 ab	-	3.5 a
4	35 b	65 b	6.2 a	5.3 a	3.4 a
5	3 c	97 a	4.5 c	4.5 a	3.5 a

Lowercase letters denote a significantly different value within the sprayed or unsprayed treatments. * Denotes statistical analysis according to Fisher’s exact test (*α* = 0.05). ** Denotes statistical analysis according to Tukey’s LSD (*α* = 0.05).

## Data Availability

The data presented in this study are available in article.
